# Radiological landmark of syndesmotic ligament complex by magnetic resonance imaging correlate with fibula free flap harvesting procedure

**DOI:** 10.1038/s41598-023-47619-2

**Published:** 2023-11-27

**Authors:** Nutcha Yodrabum, Irin Chaikangwan, Jirapat Tianrungroj, Songsak Suksantilap, Suttichai Chalalaisathaphorn, Palanan Siriwanarangsun

**Affiliations:** 1 Division of Plastic Surgery, Department of Surgery, Faculty of Medicine Siriraj Hospital, Mahidol University, Bangkok, Thailand; 2 Department of Anatomy, Faculty of Medicine Siriraj Hospital, Mahidol University, Bangkok, Thailand; 3 Department of Radiology, Faculty of Medicine Siriraj Hospital, Mahidol University, Bangkok, Thailand

**Keywords:** Bone, Ligaments, Medical research

## Abstract

Preservation of syndesmotic ligaments is crucial for preventing adverse sequelae at the donor site following free fibula osteocutaneous flap harvesting. This study sought to determine the relationship between distal tibiofibular ligaments and the fibular segment to identify radiological landmarks that facilitate safe and precise flap. The distances between the distal tibiofibular ligaments (anterior inferior tibiofibular ligament [AITFL], posterior inferior tibiofibular ligament [PITFL]) and the fibular segment, as well as the lower border of the interosseous membrane, were measured on magnetic resonance imaging (MRI) scans of 296 patients without any perceivable ankle abnormalities. The mean distances (± SD) between the distal end of the fibula and the AITFL, PITFL, and lower interosseous membrane border were 3.0 ± 0.4 cm, 2.6 ± 0.4 cm, and 3.9 ± 0.6 cm, respectively. The distance between the talar dome and the PITFL exhibited a range of 0.0–0.5 cm. Our findings support preserving a distal fibular remnant of at least 4 cm to avoid injury to the syndesmotic ligament throughout fibula osteocutaneous flap harvesting. The talar dome could serve as a useful radiological landmark for identifying the upper border of PITFL during preoperative evaluation, and thus facilitating precise and safe flap procurement.

## Introduction

The free fibular flap (FFF) technique, first conceptualized by Taylor in 1975, has since become a widely utilized method for mandibular reconstruction. By providing both osteocutaneous and vascularized bone flaps, FFF enables the efficient management of orofacial skeletal defects. It has evolved into a crucial tool for mandibular reconstruction over time, demonstrating a dependable and flexible procedure for dealing with both structural and soft tissue repair. The restoration of essential mandibular functions, such as mastication, respiration, swallowing, speech, and lip closure, is another benefit of this microvascular surgical technique in addition to its cosmetic outcomes^1,2^.

When acquiring a lengthy bone segment, the extent of the vascular pedicle presents a challenge. This can be corrected by adjusting the distal bone cutting level, thereby increasing the length of the vascular pedicle. The residual fibula length after FFF harvesting is essential for preventing postoperative complications. A 6–8 cm fibula remnant has been previously proposed to avert complications^3,4^. However, cases of ankle instability have been reported by Ganel and Yaffe^5^, indicating that the length of the fibula remnant alone may not guarantee ankle stability. Instead, ankle instability could arise from disruptions to the inferior syndesmotic ligament complex.

This ligament complex, which consists of the anterior and posterior inferior tibiofibular ligaments (AITFL and PITFL), the inferior transverse ligament, the interosseous membrane and the interosseous ligament, the latter of which is a continuation of the interosseous membrane (SDC1), protects the fibula and tibia from axial, rotational, and translational forces. Although computed tomography (CT) scans are frequently used in preoperative evaluations for FFF surgery, delivering essential information on bone structure and vascular supply, they provide limited insight into ligamentous structures. This study aims to investigate the correlation between ligamentous and bony structures in ankle magnetic resonance imaging (MRI) scans with the hypothesis that these results can be applied to CT imaging as well.

Few studies have investigated the distal tibiofibular complex's anatomy, particularly in relation to FFF surgery, whereas the majority of ankle MRI studies have focused on evaluating ligament injuries^6^. On the basis of our previous cadaveric study, a surgical landmark 2 cm above the lateral malleolus can be used intraoperatively for safe osteotomy without the potential of syndesmotic ligament injury^7^. This study aims to investigate the characteristics and relationship between the inferior syndesmotic ligaments and the fibula bone, that can be utilized for preoperative planning in FFF procedures, specifically virtual surgical planning.

## Results

A total of 296 ankle MRI scans from 296 patients were evaluated in this study. 38.7% of patients experienced chronic pain due to degenerative changes, and 36.9% of patients had a history of ankle injury. However, visible ligamentous complex injuries were not identified in any of the cases included in this study. The average patient age was 44.4 ± 12.0 years, with a mean height and BMI of 163.4 ± 9.3 cm and 25.4 ± 4.4 kg/m^2^, respectively (Table 1). Upon analyzing the results of our study, we also discovered that males exhibited greater distances between key anatomical structures than females (e.g., DF to lower border of interosseous membrane: 4.1 ± 0.6 cm vs. 3.8 ± 0.6 cm), with minimal differences between the right and left legs (e.g., DF to interosseous membrane: 4.0 ± 0.6 cm vs. 3.8 ± 0.6 cm). In general, interobserver reliability was excellent (ICC: 0.967 for DF and interosseous membrane).

**Table 1:** Characteristics of 296 patients.

Characteristics	Mean ± SD; median (IQR)		
Gender	Male (n = 111)	Female (n = 185)	Total (n = 296)
Age (years)	41.2 ± 12.5; 41.0 (30.0–53.0)	46.4 ± 11.2; 49.0 (37.0–56.5)	44.4 ± 12.0; 46 (34.0–56.0)
Height (cm)	171.9 ± 6.4; 172.0 (168.0–175.0)	158.3 ± 6.7; 158.0 (154.0–162.0)	163.4 ± 9.3; 162 (156.0–170.0)
Weight (kg)	73.9 ± 12.7; 72.0 (65.0–80.0)	64.1 ± 11.7; 63.1 (56.0–71.8)	67.8 ± 13.0; 67 (58.9–76.0)
Body mass index (kg/m^2^)	25.0 ± 4.1; 23.7 (22.4–26.9)	25.6 ± 4.5; 24.9 (22.5–28.3)	25.4 ± 4.4; 24.5 (22.4–27.8)
Right leg (n)	67	93	160
Left leg (n)	44	92	136

### Male versus female

In the comparison of gender-related differences in ankle syndesmosis system, males demonstrated a significantly greater distance than females between the ligament and bony prominence. The distances between the distal end of the fibula (DF) and the lower border of the interosseous membrane had significant difference in males and female, 4.1 ± 0.6 cm and 3.8 ± 0.6 cm, respectively (p < 0.05). Correspondingly, distances between the ligament and bony prominence were found greater in males than females. Furthermore, males exhibited significantly longer distances between the DF to the anterior inferior tibiofibular ligament (AITFL) and the DF to the posterior inferior tibiofibular ligament (PITFL) than females (AITFL: 3.2 ± 0.4 cm versus 2.9 ± 0.4 cm; PITFL: 2.7 ± 0.4 cm versus 2.5 ± 0.4 cm) (Table 2).

**Table 2:** Measurements of tibiofibular syndesmotic system characterized by gender and side of leg.

Measurements (cm, mean ± SD) (95% CI)	MRI										
	Male			Female			Total				
	Right legs (n = 67)	Left legs (n = 44)	Total (n = 111)	Right legs (n = 93)	Left legs (n = 92)	Total (n = 185)	Right legs (n = 160)	Left legs (n = 136)		Both legs (n = 296)	
Height of patients	171.9 ± 6.4			158.3 ± 6.7			163.4 ± 9.3				
BMI	25.0 ± 4.1			25.6 ± 4.5			25.4 ± 4.4				
Bony structures											
Lateral malleolus to Talar dome	2.4 ± 0.3 (2.37—2.43)	2.3 ± 0.4 (2.25—2.32)	2.4 ± 0.3** (2.27—2.36)	2.2 ± 0.3 (2.18—2.24)	2.2 ± 0.3 (2.17—2.24)	2.2 ± 0.3** (2.15- 2.23)	2.3 ± 0.3 (2.37—2.43)	2.2 ± 0.3 (2.37—2.43)		2.3 ± 0.3 (2.37—2.43)	
Fibular distal end to lateral malleolus	1.2 ± 0.4 (1.17—1.23)	1.2 ± 0.4 (1.16—1.22)	1.2 ± 0.4** (1.15—1.23)	0.9 ± 0.4 (0.87—1.03)	0.9 ± 0.2 (0.88- 1.03)	0.9 ± 0.3** (0.88- 1.10)	1.0 ± 0.4 (0.97–1.14)	1.0 ± 0,3 (0.98–1.15)		1.0 ± 0.4 (0.97–1.14)	
Fibular distal end to Talar dome	2.9 ± 0.4 (2.88—2.92)	3.0 ± 0.4 (2.98–3.02)	2.8 ± 0.3 (2.78–2.83)	2.6 ± 0.4 (2.57–2.62)	2.9 ± 0.3 (2.88–2.92)	2.8 ± 0.3 (2.88–2.93)	2.7 ± 0.5 (2.65–2.74)	2.8 ± 0.6 (2.77–2.83)		2.9 ± 0.6 (2.88–2.95)	
Interosseous ligament/interosseous membrane (IL/IM)											
Talar dome to IM lower end (IL)	1.7 ± 0.6 (1.67–1.73)	1.7 ± 0.6 (1.67–1.73)	1.7 ± 0.6 (1.67–1.73)	1.6 ± 0.5 (1.56–1.62)	1.6 ± 0.6 (1.56–1.63)	1.6 ± 0.5 (1.57–1.62)	1.7 ± 0.5 (1.67–1.72)	1.6 ± 0.6 (1.58–1.63)		1.7 ± 0.5 (1.67–1.73)	
Fibular distal end to IM lower end (IL)	4.2 ± 0.6 (4.16–4.24)	4.1 ± 0.6 (4.07–4.13)	4.1 ± 0.6 (4.18–4.23)	3.8 ± 0.5 (3.71–3.79)	3.8 ± 0.6 (3.75–3.82)	3.8 ± 0.6 (3.79–3.86)	4.0 ± 0.6 (3.94–4.11)	3.9 ± 0.6 (3.87–3.93)		3.9 ± 0.6 (3.87–3.93)	
Lateral malleolus to IM lower end (IL)	3.1 ± 0.3 (3.07–3.13)	2.9 ± 0.7 (2.86–2.92)	3.0 ± 0.7 (2.98–3.02)	2.9 ± 0.6 (2.88–2.92)	2.8 ± 0.6 (2.75–2.81)	2.8 ± 0.6 (2.76—2.81)	3.0 ± 0.6 (2.97–3.03)	2.8 ± 0.6 (2.78 –2.82)		2.9 ± 0.6 (2.87–2.92)	
Anterior inferior tibiofibular ligament (AITFL)*											
Talar dome to AITFL	0.7 ± 0.4 (0.67–0.73)	0.8 ± 0.4 (0.77–0.82)	0.8 ± 0.4 (0.77–0.82)	0.7 ± 0.4 (0.67–0.73)	0.7 ± 0.3 (0.68–0.72)	0.7 ± 0.4 (0.67–0.73)	0.8 ± 0.4 (0.77–0.82)	0.7 ± 0.4 (0.68–0.72)		0.7 ± 0.4 (0.68–0.72)	
Fibular distal end to AITFL	3.2 ± 0.4 (3.16–3.21)	3.1 ± 0.5 (3.10–3.15)	3.2 ± 0.4** (3.17–3.21)	2.8 ± 0.4 (2.77–2.83)	2.9 ± 0.4 (2.85–2.89)	2.9 ± 0.4** (2.87–2.91)	3.0 ± 0.5 (2.99–3.04)	3.0 ± 0.4 (2.98–3.02)		3.0 ± 0.4 (2.98–3.02)	
Lateral malleolus to AITFL	2.0 ± 0.6 (1.95–2.01)	2.1 ± 1.2 (2.02–2.13)	2.0 ± 0.6 (1.97–2.03)	2.0 ± 0.5 (1.97–2.02)	1.9 ± 0.5 (1.87–1.93)	2.0 ± 0.5 (1.97–2.02)	2.0 ± 0.5 (1.98–2.03)	2.0 ± 0.5 (1.98–2.03)		2.0 ± 0.5 (1.98–2.03)	
Posterior inferior tibiofibular ligament (PITFL)*											
Talar dome to PITFL	0.4 ± 0.6 (0.38–0.42)	0.5 ± 0.8 (0.45–0.53)	0.4 ± 0.7 (0.38–0.43)	0.3 ± 0.3 (0.27–0.31)	0.3 ± 0.3 (0.27–0.31)	0.3 ± 0.3 (0.27–0.31)	0.3 ± 0.5 (0.27–0.32)	0.4 ± 0.5 (0.38–0.43)		0.3 ± 0.5 (0.27–0.32)	
Fibular distal end to PITFL	2.7 ± 0.4 (2.68–2.74)	2.7 ± 0.4 (2.68–2.73)	2.7 ± 0.4** (2.68–2.73)	2.5 ± 0.4 (2.48–2.72)	2.4 ± 0.4 (2.36–2.41)	2.5 ± 0.4** (2.48–2.72)	2.6 ± 0.4 (2.58–2.62)	2.5 ± 0.4 (2.45–2.5)		2.6 ± 0.4 (2.58–2.62)	
Lateral malleolus to PITFL	1.5 ± 0.5 (1.47–1.53)	1.6 ± 0.4 (1.58–1.62)	1.5 ± 0.5 (1.48–1.53)	1.5 ± 0.4 (1.46–1.51)	1.5 ± 0.4 (1.47–1.51)	1.5 ± 0.4 (1.47–1.51)	1.5 ± 0.5 (1.47–1.52)	1.5 ± 0.4 (1.48–1.52)		1.5 ± 0.4 (1.48–1.52)	

*Upper end.

**Significant difference (*p* < 0.05).

### Right leg versus left leg

There appears to be no statistically significant difference (p < 0.05) between the right and left legs regarding the examined anatomical structures. In the right and left legs, the distance between the distal end of the fibula (DF) and the lower border of the interosseous membrane were 4.0 ± 0.6 cm and 3.8 ± 0.6 cm, respectively. The distances between DF and AITFL were similar in both right and left legs (3.0 ± 0.4 cm), as were the distances between DF and PITFL in the right and left legs (2.6 ± 0.4 cm and 2.5 ± 0.4 cm) (Table 2).

Regarding the lateral aspect, the prominence of the lateral malleolus was located 1.0 ± 0.4 cm and 1.0 ± 0.3 cm respectively, from the DF in the right and left legs. Both the right (2.3 ± 0.3 cm) and left (2.2 ± 0.3 cm) legs had virtually identical distances between the DF and the talar dome. Likewise, the distance between the lateral malleolus and talar dome in the right and left legs was 1.3 ± 0.4 cm and 1.3 ± 0.3 cm, respectively (Table 2).

Regardless of the patient's gender or leg side, the distance between PITFL and the talar dome ranged from 0 to 0.5 cm, with a median of 0.3 cm (Table 2).

### Interobserver reliability

The interobserver reliability for measuring the distance between the distal end of the fibula (DF) and the lower border of the interosseous membrane in the right and left legs of all readers were excellent, with an intraclass correlation coefficient (ICC) of 0.967 (95% CI 0.930–0.984). Other distance measurements ranged from poor to excellent interobserver reliability (ICC: 0.486–0.997). With a kappa value of 1.00, the shape interobserver reliability demonstrated perfect agreement.

### Regression analysis

According to Table 3 regression analysis, each patient's position of the ligament is influenced by their height and/or gender. With respect to the anterior inferior tibiofibular ligament (AITFL), for each 1 cm height increment, the distance between the distal end of the fibula (DF) and AITFL increases by 0.008 cm, and by 0.190 cm for male patients. Moreover, for every 1 cm increase in height, the distance between the DF and the posterior inferior tibiofibular ligament (PITFL) enlarges by 0.016 cm (p < 0.01). Lastly, the distance between the DF and the lower border of the interosseous membrane expands by 0.022 cm for each 1 cm height increment.

**Table 3:** Multiple linear regression of patients’ characteristic and distance between syndesmotic ligament complex and radiological bony anatomy.

Parameter	Constant		Sex (female [0] and male [1])			Side of legs (right [0] and left [1])		Age (years)		Height (cm)			Weight (kg)		
	B0	p-value	B1		p-value	B2	p-value	B3	p-value	B4		p-value	B5		p-value
Fibular distal end to IM lower end	0.360	0.524	–		–	–	–	–	–	0.022*		< 0.010	–		–
Talar dome to AITFL	− 0.184	0.650	–		–	–	–	–	–	0.006*		0.023	–		–
Fibular distal end to AITFL*	1.390	0.012	0.190*		< 0.010	–	–	–	–	0.008*		0.032	–		–
Lateral malleolus to AITFL	1.984	< 0.01	–		–	–	–	–	–	–		–	–		–
PITFL to talar dome	− 1.338	< 0.01	–		–	–	–	–	–	0.010*		< 0.010	–		–
Fibular distal end to PITFL*	− 0.047	0.897	–		–	–	–	–	–	0.016*		< 0.010	–		–
Lateral malleolus to PITFL	0.147	0.746	–		–	–	–	–	–	0.008*		< 0.010	–		–

*Significant difference (*p* < 0.05).

*AITFL* anterior inferior tibiofibular ligament, *PITFL* posterior inferior tibiofibular ligament.

## Discussion

Our research illustrates the importance of preserving the syndesmotic ligament complex to avoid post-operative ankle instability. Prior studies, such as those by Littlechild et al.^8^ and Pacelli et al.^9^, have demonstrated that injury to the syndesmotic ligaments can alleviate talar shift and ankle instability.

The interosseous ligament has been thoroughly examined in our work as a crucial element of the syndesmotic complex, a well acknowledged observation supported by numerous anatomical sources. The interosseous ligament is located at the distal end of the interosseous membrane and is acknowledged as a crucial stabilizer of the distal tibiofibular syndesmosis^10,11^. During our measuring technique, we have designated 'the lower end of the interosseous membrane' as a point of reference, which inherently encompasses the interosseous ligament by virtue of its physical location. The significance of the interosseous ligament in the preservation of ankle stability and the prevention of proximal migration of the talus is duly recognized. Our methodology ensured that the interosseous ligament, while not expressly mentioned in every instance within our publication, was considered in our evaluations. The thorough incorporation of the lower interosseous membrane, and therefore the interosseous ligament, serves as a fundamental basis for our examination of the syndesmotic complex and lends credibility to our research outcomes. Moreover, with the inclusion of the distal portion of the interosseous membrane in our measurements, we were able to comprehensively capture the functional anatomy of the syndesmosis in a manner that is clinically significant. This is crucial for ensuring precise diagnosis and facilitating effective surgical planning.

Using ankle magnetic resonance imaging, we measured the distances between the distal end of the fibula and the proximal ends of the AITFL and PITFL. These distances were found to be approximately 3.0 ± 0.4 cm and 2.6 ± 0.4 cm, respectively. Our results indicate that an approximate distal fibular remnant minimum length of 4 cm is required to protect the syndesmotic ligaments. These measurements are applicable to Thai patients undergoing fibular flap harvesting procedures.

In situations where patients present with extensive mandibular defects, a longer fibular free flap is typically required to achieve optimal reconstructive outcomes. Differing from previous studies which suggested a fibular remnant length of 6–8 cm, our study has identified that a shorter fibular remnant length (4 cm) can also be effective, representing a significant advancement in surgical technique. This discovery permits clinicians to utilize a larger portion of the fibula for the free flap, thereby facilitating improved operative and reconstructive outcomes in cases involving complex or large mandibular defects. Integrating these insights, surgeons can tailor their approach to the specific needs of each patient, thereby improving the efficacy of mandibular reconstruction and the restoration of crucial functional and aesthetic parameters.

Our outcomes are in line with previous research. For instance, Pacelli et al. discovered that maintaining syndesmotic ligament stability and preventing ankle instability required leaving 10% of the fibular shaft (3.9 cm) preserved^11^. Additionally, Ongsiriporn et al. reported comparable distances between the distal end of the fibula and the AITFL and PITFL, suggesting that the lateral malleolus could function as a surgical landmark for fibula harvesting^7^.

Multiple linear regression analyses were utilized to determine ligament length predictors. The most reliable predictor of the distance between the distal end of the fibula and the AITFL, according to the regression analysis presented in Table 3, was the patient's height. Specifically, the distance between the DF and AITFL increased by 0.008 cm for every 1 cm increase in height, with male patients exhibiting a greater distance of 0.190 cm than female patients. These results indicate that height is a more accurate predictor of the distance between the DF and AITFL than gender. Nevertheless, individualized data facilitates more precise ligament level estimation, thereby decreasing the risk of iatrogenic ligament injury and subsequent complications.

Our study also indicates that the proximal portion of the PITFL is located slightly above the talar dome, with a mean distance of 0.3 cm (ranging from 0 to 0.5 cm) and in an almost identical location. This result has implications for the use of the talar dome as a radiological landmark for the upper PITFL boundary. Further study is warranted into the idea that distal fibula osteotomies shouldn't go past the tibial epiphyseal plate to prevent disruption of the anterior tibiofibular ligament (AITFL).

This study has several clinical implications for fibula free flap surgeons, particularly regarding MRI-to-CT knowledge transfer. Additionally, we can inform CT-guided surgical planning by examining the ligament-bone relationship in MRI scans. The previous study^7^ suggests that CT scans and the surgical landmark 2 cm above the lateral malleolus can be used for a safe osteotomy without syndesmotic ligament injury. Despite poor ligament visualization on CT scans, surgeons can use this knowledge to choose the best osteotomy site.

Our study emphasizes preserving the syndesmotic ligament complex during fibula free flap harvesting. Surgeons can use CT scans for preoperative planning and reduce ankle instability by understanding MRI anatomical relationships.

In conclusion, our study demonstrates that the knowledge gained from MRI scans regarding the relationship between the inferior syndesmotic ligaments and the fibula bone can be applied to CT scans for preoperative planning in fibula free flap surgery. By taking these findings into account, surgeons can improve patient outcomes and reduce the risk of postoperative complications, even when using CT scans that have limitations in displaying ligamentous structures.

### Limitations

Our study emphasis on the relationship between the inferior syndesmotic ligaments and fibula bone in free fibular flap (FFF) surgery, but it still has limitations. First, demographic diversity may not be representative of the international population.

Lastly, the inferior tibiofibular ligament, also known as the deep portion of the PITFL, was excluded from our study due to the small size of its fibers, which made identification with MRI challenging. Our investigation into the relation between the inferior syndesmotic ligaments and the fibula bone may be limited by the exclusion of this ligament^8,9^.

These limitations should be considered when interpreting and implementing the results in clinical practice. Future research should seek to address these limitations, possibly by conducting prospective, multicenter studies with larger, more diverse patient populations and by investigating additional factors that may affect the surgical outcome.

## Material and methods

### Study population and demographics

This retrospective study involved a thorough review of MRI scans at Siriraj Hospital, conducted between January 2016 and May 2022. A total of 350 patients' MRI scans were initially considered. A highly experienced board-certified radiologist specializing in musculoskeletal imaging rigorously examined each scan, ensuring the exclusion of any pathological findings. Post this stringent revision, after applying exclusion criteria such as prior ankle pathology or surgery, compromised image quality, and systemic diseases affecting bone health, A total of 54 patients were excluded from our study. Consequently, the study ultimately included 296 ankle MRI scans from 296 patients.

The demographic composition of our study population was predominantly Thai, comprising 98% of the total subjects. The remaining 2% were divided equally between Caucasians and other ethnicities, thus maintaining a diverse representation. The gender distribution consisted of 185 women and 111 men, and the scans included a balanced representation of 160 right and 136 left ankles. All scans were performed on either a 1.5 or 3.0 Tesla MRI machine.

### MRI scanning protocol

All MR studies of the ankles were done using 3.0 T MR machines, including two Ingenia 3.0 T; Philips Healthcare, Best, Netherlands (installed in 2013 and 2014); and one MAGNETOM vida 3.0 T; Siemens, Erlangen, Germany (installed in 2019). Phased array ankle coils were also applied to the ankle. For conventional MR images, patients were imaged supine with the toe up position. The scanning parameters were listed as following; Coronal proton density image (PD): TR = 2900 mS, TE = 30 mS, FOV 220 × 160 mm, matrix 512 × 384; Coronal T2W: TR = 3450 mS, TE = 80 mS, FOV 220 × 160 mm, matrix 512 × 384; Coronal T2WFS: TR = 3700 mS, TE = 80 mS, FOV 210 × 150 mm, matrix 512 × 384; Axial T2W; TR = 3200mS, TE = 80, FOV 210 × 150 mm, matrix 512 × 256; Axial T2WFS; TR = 3600mS, TE = 80, FOV 210 × 150 mm, matrix 512 × 256. All sequences were obtained in 3-mm slice thickness with minimal space (0.3 mm).

### Measurement team composition and procedure

Our measurement team, comprising two radiologists and one plastic surgeon, initially reached a consensus on the syndesmotic anatomy and the precise methodology for measuring each ligament on MRI. This understanding was validated by an expert radiologist to ensure accuracy and consistency across the team. To calibrate our measurement approach, we utilized a set of 30 ankle MRI scans for a pilot test. Each team member measured the same 30 MRI scans to identify any potential issues and to standardize the measurement technique. Following this pilot phase, if any problematic measurements arose, in scenarios where measurement discrepancies exceeded 2 mm, additional meetings were convened to achieve a consensus and refine the measurement protocol, thereby minimizing errors.

Each observer then measured each scan three times, with the average of these three measurements being recorded as the final data point for that scan. To ascertain the precision of each observer's measurements, intraobserver reliability was calculated, yielding coefficients between 0.83 and 0.94. Furthermore, to evaluate the consistency across different observers, interobserver reliability was assessed using the Intraclass Correlation Coefficient (ICC) with a two-way mixed absolute agreement model. This yielded highly accurate results.

Following the successful completion of the pilot test, all three readers were deemed validated to participate in the data collection of the remaining 296 MRI scans. The scans were distributed evenly among the readers, with reader 1 assigned 99 scans, reader 2 another 99 scans, and reader 3 the final 98 scans. Each reader conducted four separate readings of each assigned MRI at different time intervals to prevent recall bias. The average of these four readings was then calculated and used as the final measurement for each scan.

The workflow of our study, encompassing various stages and methodologies employed, is comprehensively illustrated in the diagram provided in the Supplementary Digital Content (SDC2).

### Measurement procedure

**Figure 1:**
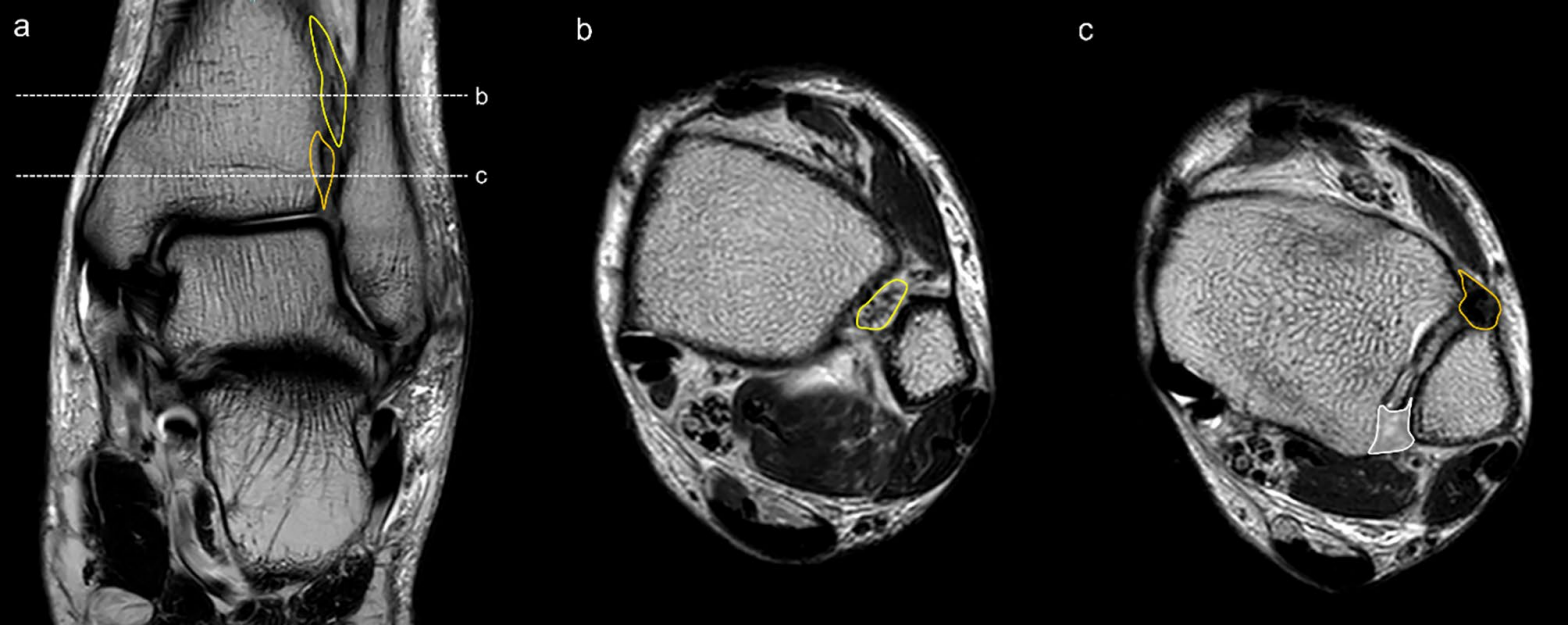
(**a**) The coronal view of the ankle, with axial view cuts depicted at the mid-interosseous ligament level (**b**) and at the middle of anterior tibiofibular ligament (**c**). (**b**) An axial view of the ankle with the interosseous ligament highlighted in yellow. (**c**) An axial view of the ankle with the anterior tibiofibular ligament and posterior tibiofibular ligament highlighted in orange and white, respectively.

**Figure 2:**
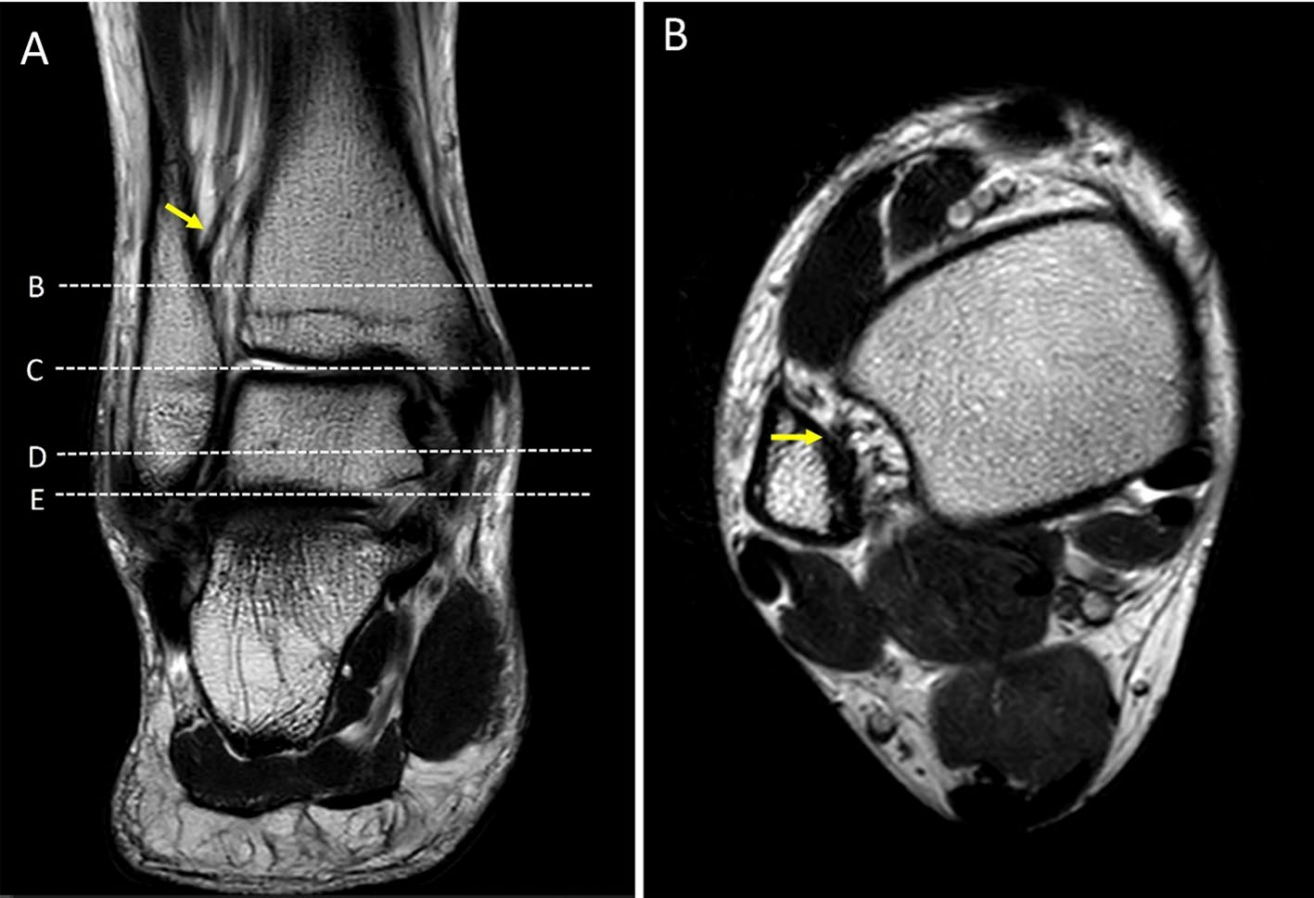
Coronal T2W of the ankle (**A**); the first dot-line-B indicates level of the inferior border of the interosseous ligament (yellow arrow). Dot-line-C: level of the talar dome. Dot-line-D: level of lateral malleolus. Dot-line-E: most distal part of the lateral malleolus/fibular. Axial T2W of the ankle at level of the inferior border of the interosseous ligament (**B**) demonstrates the interosseous ligament (yellow arrow).

**Figure 3:**
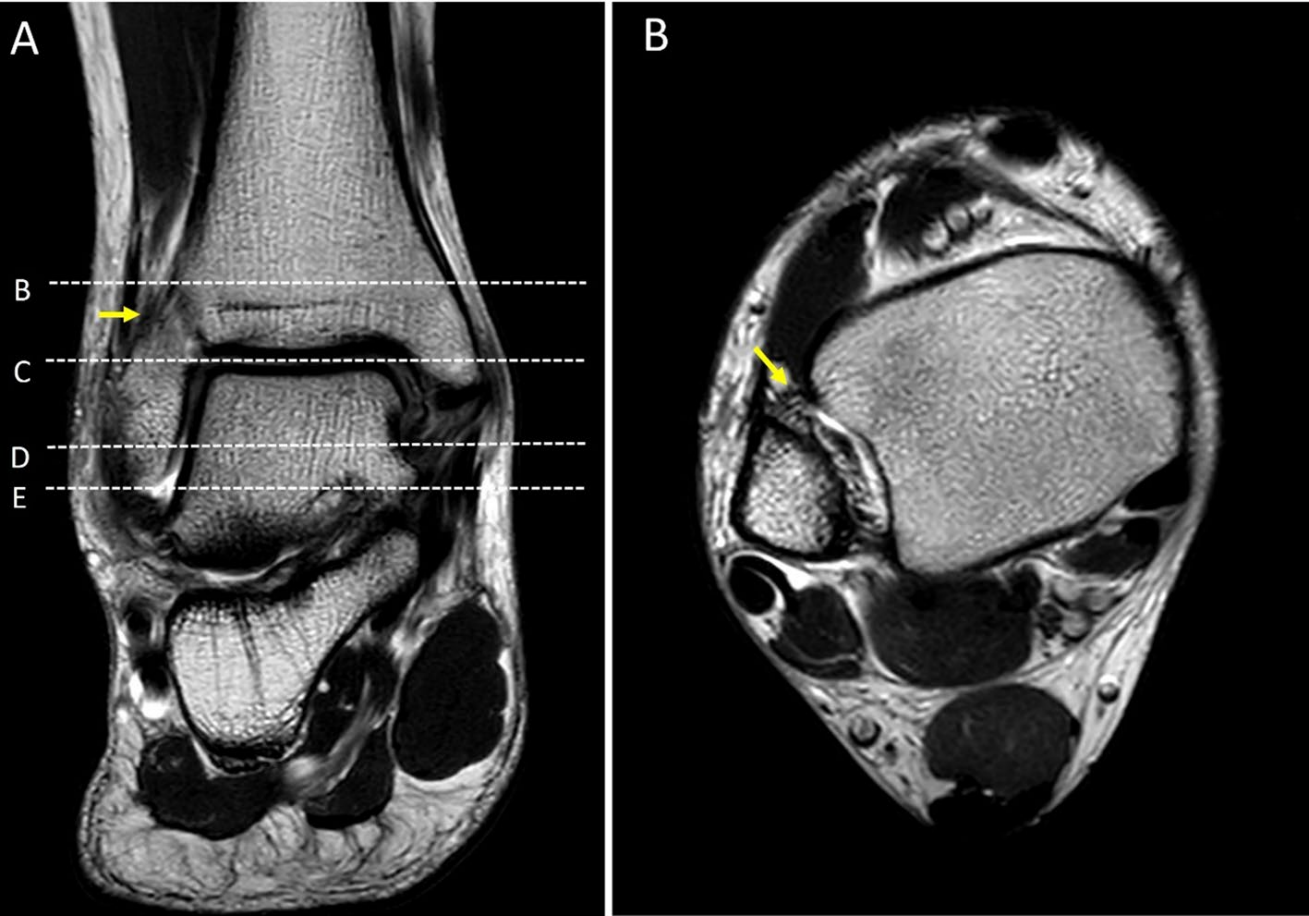
Coronal T2W of the ankle (**A**); the first dot-line-B indicates level of the proximal part of the anterior inferior tibiofibular ligament (AITFL; yellow arrow). Dot-line-C: level of the talar dome. Dot-line-D: level of lateral malleolus. Dot-line-E: most distal part of the lateral malleolus/fibular. Axial T2W of the ankle (**B**) at level of the proximal part of the AITFL (yellow arrow).

**Figure 4:**
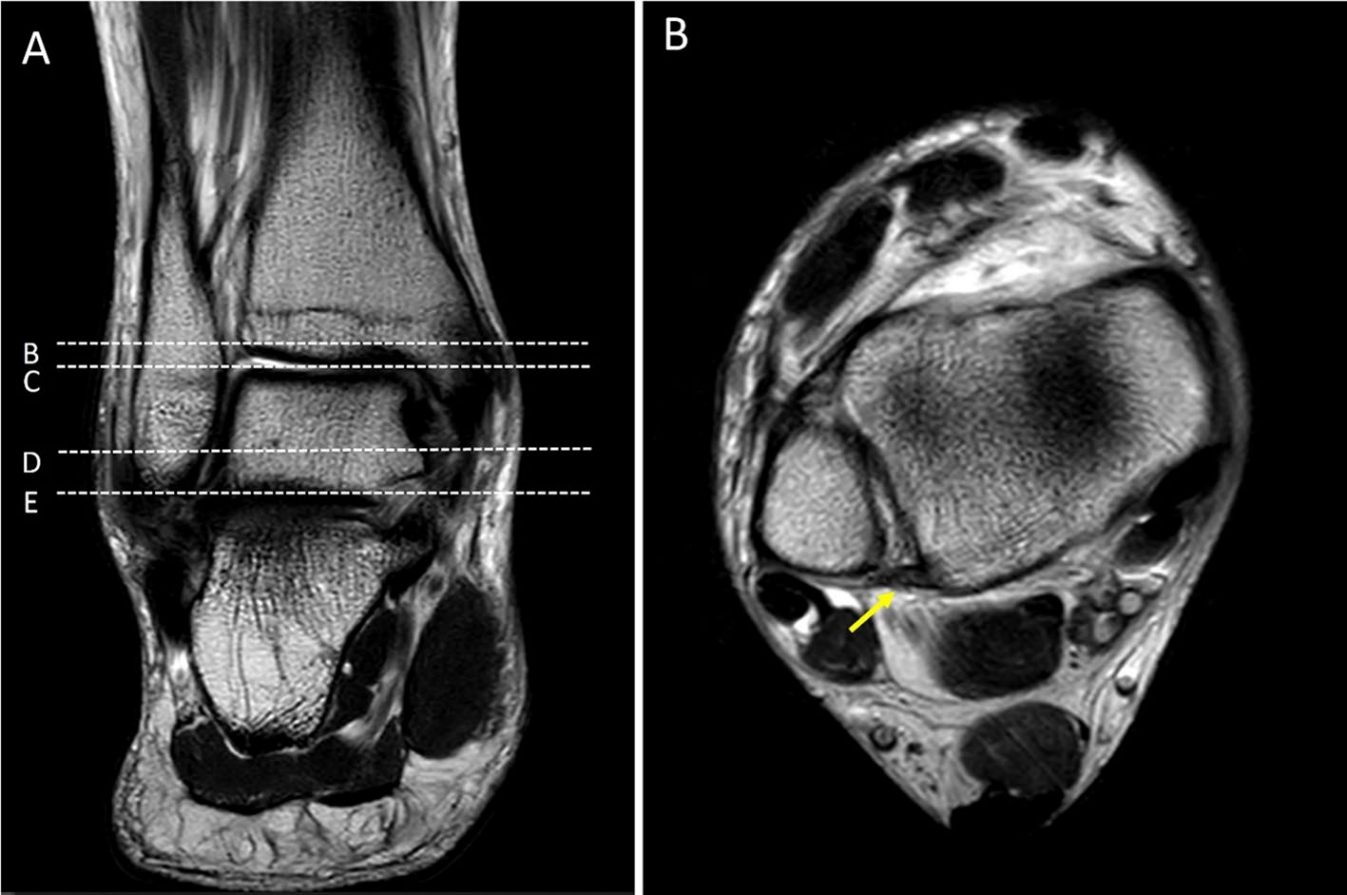
Coronal T2W of the ankle (**A**); the first dot-line-B indicates level of the posterior inferior tibiofibular ligament (PITFL). Dot-line-C: level of the talar dome. Dot-line-D: level of lateral malleolus. Dot-line-E: most distal part of the lateral malleolus/fibular. (**B**) Axial T2W of the ankle at level of the PITFL (yellow arrow).

**Figure 5:**
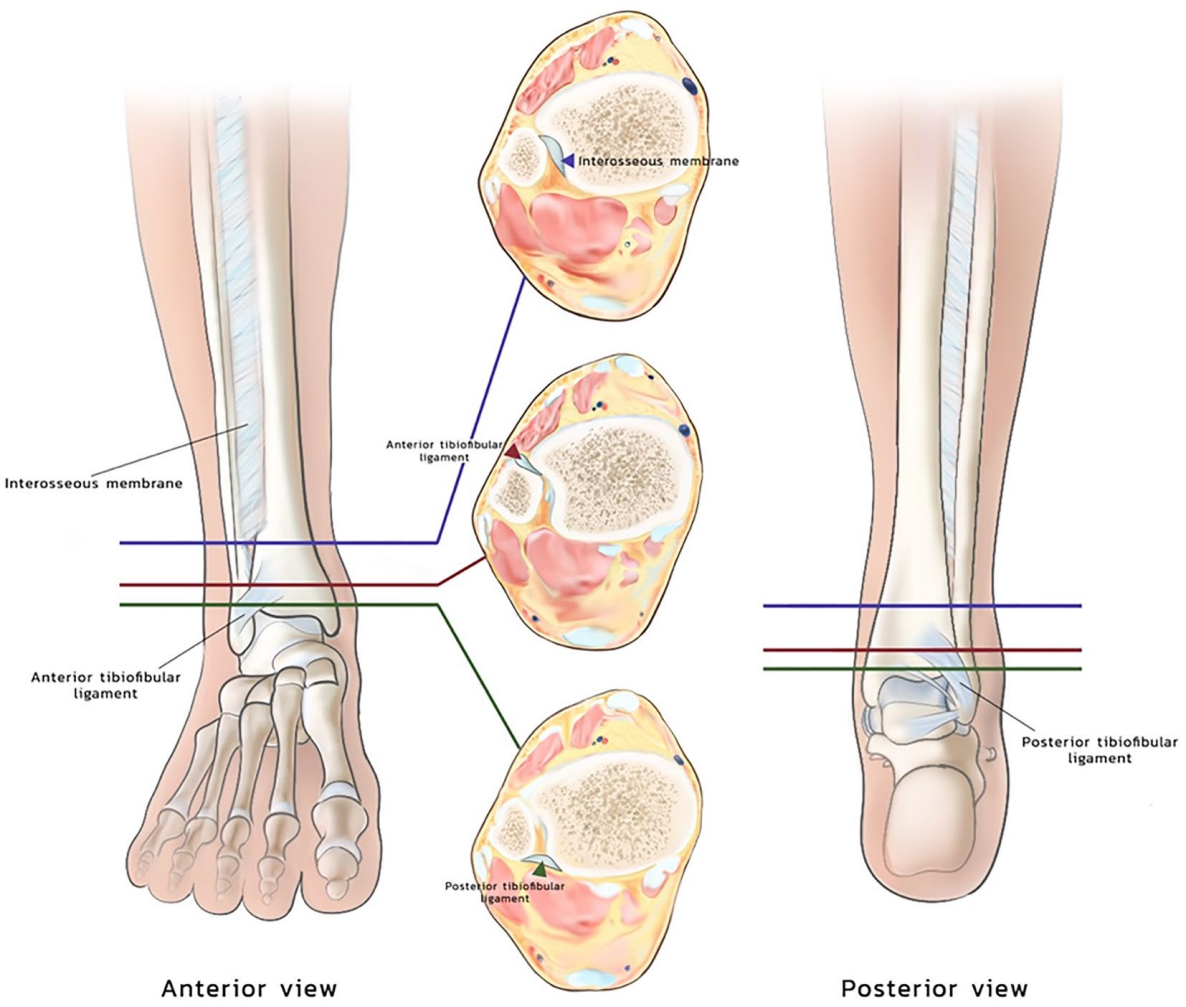
Illustration of lower border of interosseous ligament, proximal part of AITFL and proximal part of PITFL in both axial and coronal view.

The inferior syndesmotic ligament was identified on MRI scans, with measurements conducted in both axial and coronal views. Our methodology leveraged the reference points demonstrated in Figs. 1, 2, 3, 4, and 5. These reference points included the talar dome, lateral malleolus, distal end of the fibula, interosseous ligament which located at the lower end of the interosseous membrane, and the uppermost portion of the syndesmotic ligaments, specifically the anterior inferior tibiofibular ligament (AITFL) and posterior inferior tibiofibular ligament (PITFL). The work of Lee et al.^12^ influenced this method by attempting to visualize the entire ligament in multiple planes, including axial, axial oblique, coronal, and sagittal. In addition, Brown et al.^13^ determined that the optimal perspective for evaluating the AITFL is the axial plane.

### Statistical analysis

Statistical evaluations were performed using IBM SPSS Statistics for Windows, version 25.0 (Armonk, NY: IBM Corp). The normality of data distribution was assessed, followed by t-tests to compare group differences. Multiple linear regression analyses were used to explore the relationship between syndesmotic ligament complex distances and demographic variables. Insignificant variables were removed using backward elimination, with a statistical significance cutoff set at p < 0.05.

This study was approved by the Siriraj Hospital's Ethics Committee (Certificate of Approval no. Si 451/2021; SIRB Protocol no. 163/2564), adhering to all relevant guidelines and regulations. Informed consent was obtained from all subjects or their legal guardians.

## Conclusion

Our study, of all radiological review of Thai patients, offers valuable insights into the optimal length of the distal fibular remnant during fibula osteocutaneous flap harvesting. Our findings suggest preserving a distal fibular remnant of at least 4 cm is sufficient to prevent injury to the syndesmotic ligament, in contrast to earlier recommendations for a 6–8 cm remnant. Moreover, the talar dome has been identified as a useful radiological landmark for determining the upper border of PITFL during intraoperative evaluation, thereby facilitating accurate and safe flap procurement.

### Supplementary Information

Supplementary Legends.

Supplementary Information 2.

Supplementary Information 3.

## Data Availability

The datasets generated during and/or analyzed during the current study are available from the corresponding author on reasonable request.
