# Supplementary material for: Radiological landmark of syndesmotic ligament complex by magnetic resonance imaging correlate with fibula free flap harvesting procedure
Source: Sci Rep. 2023 Nov 27;13:20844. doi: 10.1038/s41598-023-47619-2 (PMC10682006; doi:10.1038/s41598-023-47619-2)
Supplement: Supplementary file 1 — Supplementary Legends. [file 41598_2023_47619_MOESM1_ESM.docx]

**SDC Legends**

**SDC1)** Anatomical Landmark Identification in Ankle MRI for Syndesmotic Ligament Measurement.
White Dashed Line: Indicates the most inferior portion of the interosseous ligament.
Yellow Dashed Line: Represents the level of the talar dome/tibiotalar joint.

Orange Dashed Line: Marks the level of the lateral malleolus.

Red Dashed Line: Denotes the level of the tip of the fibula.

**SDC2)** Standardized Measurement Workflow for Ankle MRI Evaluation in Syndesmotic Ligament Study:

This diagram illustrates the comprehensive process undertaken by our team to standardize and validate the measurement of syndesmotic ligaments in ankle MRIs, including a pilot test for calibration, intra- and interobserver reliability assessments, and the final measurement protocol for data collection.
