# Supplementary figures and images for: Radiological landmark of syndesmotic ligament complex by magnetic resonance imaging correlate with fibula free flap harvesting procedure
Source: Sci Rep. 2023 Nov 27;13:20844. doi: 10.1038/s41598-023-47619-2 (PMC10682006; doi:10.1038/s41598-023-47619-2)

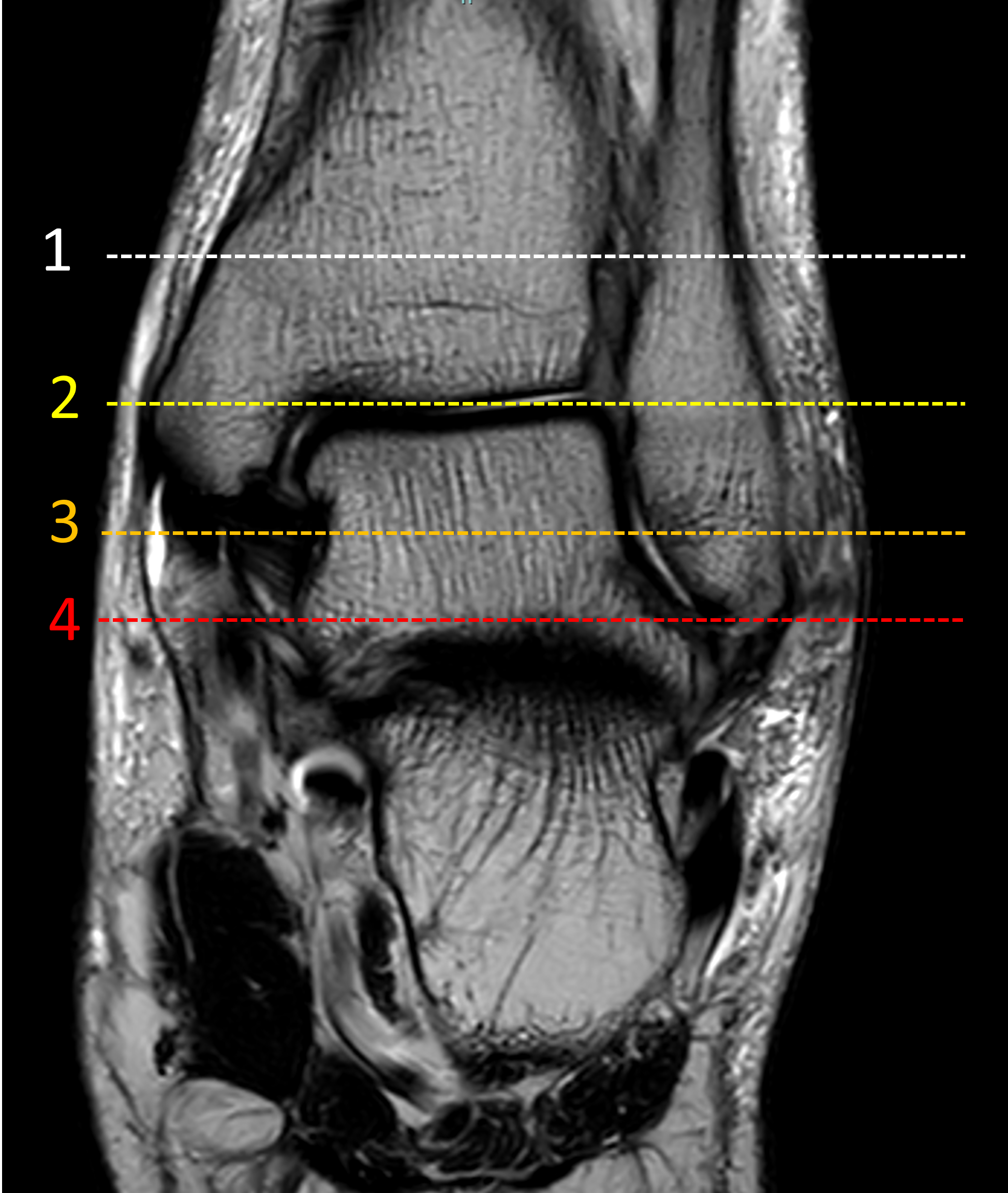

Supplement: Supplementary file 2 — Supplementary Information 2. [file 41598_2023_47619_MOESM2_ESM.tif]

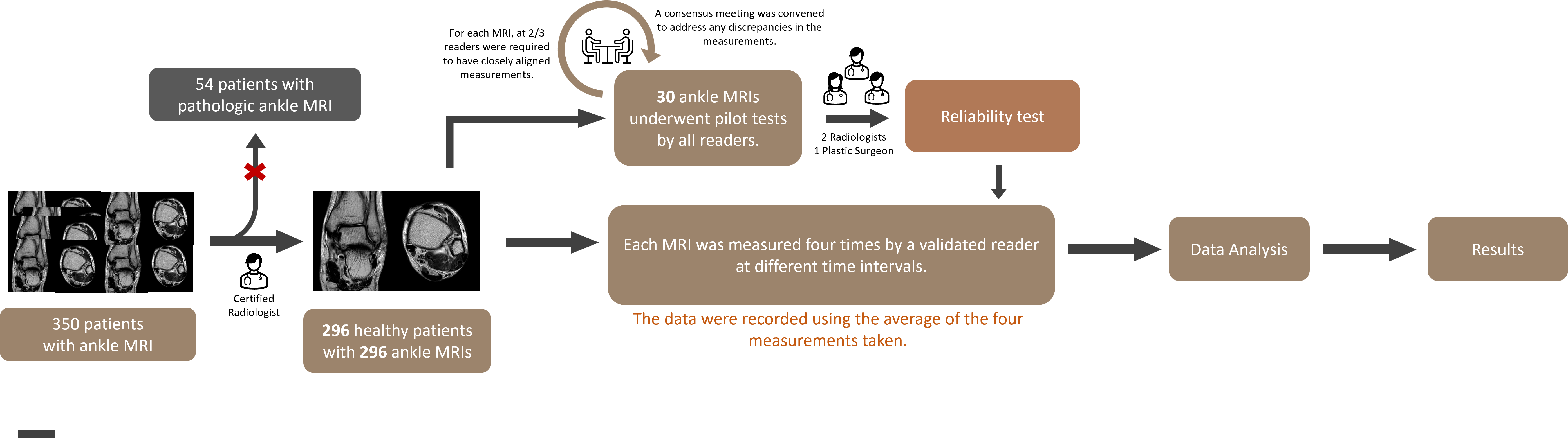

Supplement: Supplementary file 3 — Supplementary Information 3. [file 41598_2023_47619_MOESM3_ESM.tif]
